# Illegitimate translation causes unexpected gene expression from on-target out-of-frame alleles created by CRISPR-Cas9

**DOI:** 10.1038/srep39608

**Published:** 2016-12-21

**Authors:** Shigeru Makino, Ryutaro Fukumura, Yoichi Gondo

**Affiliations:** 1 Mutagenesis and Genomics Team, RIKEN BioResource Center, 3-1-1 Koyadai, Tsukuba, Ibaraki, 305-0074, Japan

## Abstract

CRISPR-Cas9 is efficient enough to knock out both alleles directly by introducing out-of-frame mutations. We succeeded in making biallelic on-target frameshift mutations of the endogenous *Gli3* gene; however, the GLI3 protein was expressed in all six of the established cell lines carrying homozygous out-of-frame mutations. We developed a dual-tagged expression vector and proved that illegitimate translation (ITL) was the cause of the unexpected *Gli3* expression. Thus, gene expression must be examined even if designed on-target out-of-frame sequences are introduced by genome editing. In addition, it is highly recommended to pre-examine the occurrence of ITL *in vitro* prior to the design and construction of any genome-editing vectors. *In vitro* assay systems such as the dual-tagged ITL assay system developed in this study should aid the identification and elucidation of ITL-based human diseases and gene expression.

Genome editing technologies have made it possible to primarily construct frameshift mutations by efficiently introducing double-strand breaks (DSBs) to the target site in the genome^1^,^2^,^3^. The out-of-frame mutations are then expected to produce null or knockout alleles due to the early appearance of stop codons. However, in nonsense mutations near the 5′ region of open reading frames (ORFs), translation can be initiated from an in-frame ATG other than the authentic translation initiation codon. Such illegitimate translation (ITL) proteins have been reported as a genetic factor associated with human diseases^4^,^5^,^6^,^7^. Moreover, ITL has been found in normal genes without mutations; for example, upstream open reading frames (uORFs) regulate gene expression in response to environmental conditions^8^,^9^. Thus, out-of-frame mutations established by genome editing may result in protein products due to ITL.

Using the CRISPR-Cas9 system^10^,^11^, we have established cell lines carrying expected frameshift mutations of the target gene. We found that all of the biallelic out-of-frame mutations expressed the target gene product(s) due to ITL. We emphasize the importance of validating gene products even if the designed mutation is introduced by genome editing. We also developed an *in vitro* assay system for ITL that may be useful for genome editing and studying the molecular mechanisms of ITL.

## Results and Discussion

**Figure 1:**
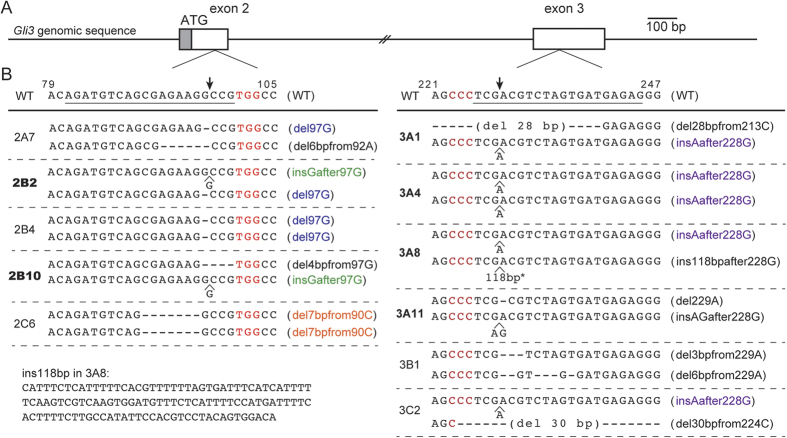
Establishment of *Gli3* mutant cell lines. (**A**) Exon-intron structure of the mouse *Gli3* gene. Exon2 contains the translation initiation codon. (**B**) A part of the *Gli3* sequences with the target sites of DSBs (arrows) by Cas9 nuclease. Target sequences for sgRNA are underlined, and PAMs are shown in red. Numerals over the WT sequence indicate the nucleotide position in the ORF sequence from the first base of the start codon (+1). Identical clones are indicated by the colored allele names in parentheses. The six clones with bold letters having biallelic out-of-frame mutations were subjected to Western analysis.

We performed genome editing to knock out the *Gli3* gene in mouse NIH3T3 cells using the CRISPR-Cas9 system^10^,^11^ to elucidate Hedgehog (Hh) signaling. *Gli3* has 15 exons. To obtain complete null alleles, we introduced DNA double-strand break/non-homologous end joining (DSB/NHEJ) to either exon 2 or exon 3 (Fig. 1A) of the *Gli3* gene. We established 11 cell lines, all of which carried biallelic insertion-deletion (indel) mutations at the targeted DSB/NHEJ sites on either exon 2 or exon 3 (Fig. 1B). Among 22 independent indel alleles, 9 unique frameshift alleles were obtained by eliminating identical mutations.

**Figure 2:**
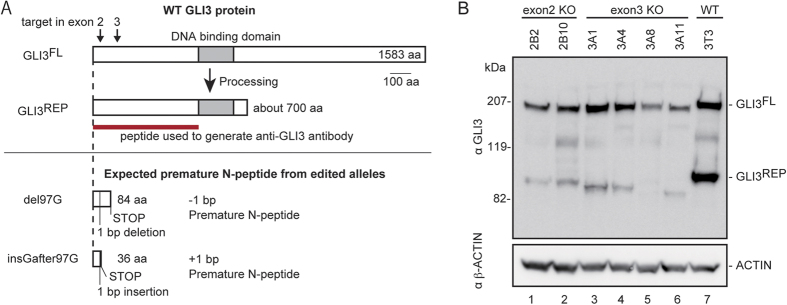
Expression of unexpected GLI3 protein from the frameshifted *Gli3* alleles *in vivo*. (**A**) Schematic representation of WT and expected premature GLI3 proteins. When Hh signaling is repressed, it is known that the transcriptional activator of WT GLI3 (GLI3^FL^) is proteolytically processed to the repressor form of GLI3^REP13^. The anti-GLI3 antibody used in this study was generated against the N-terminal half of human GLI3 shown in the red bar. (**B**) Western blotting of lysates from WT (NIH3T3) and the six mutant cell lines carrying biallelic frameshift mutations with anti-GLI3 antibody.

The six cell lines carried out-of-frame indel mutations in both alleles; thus, they were the homozygous null candidates (Fig. 1B). We confirmed that NIH3T3 cells carried only two *Gli3* alleles without any hyperdiploidy (Figures S1A and B). The six cell lines were expected either to express nonfunctional premature N-peptides (Fig. 2A) or to completely lack protein products due to nonsense-mediated mRNA decay^12^. To examine GLI3 expression, lysates from the cell lines were subjected to Western blot analysis. Notably, the GLI3 protein is known to undergo post-translational modification^13^. The C-terminal half of full-length GLI3 (GLI3^FL^) is occasionally degraded to generate GLI3^REP^ through a proteasome-mediated process (Fig. 2A). GLI3^REP^ functions as a transcriptional repressor of Hh signaling^13^. As shown in Fig. 2B, two signals for GLI3^FL^ (1583 amino acids (aa)) and GLI3^REP^ (~700 aa) were observed in the wild-type (WT) cells, as expected from the post-translational modification.

Unanticipated GLI3 proteins were observed in all of the six mutant cell lines. The size range of the expected premature N-peptide was estimated to be between 36 and 117 aa residues (see Fig. 2A and S2–S5). Notably, β-ACTIN consists of 375 aa; thus, the Western blot signals of the premature N-peptides would be much smaller than that of β-ACTIN in Fig. 2B. We never detected signals smaller than β-ACTIN (Figures S6A and B), probably because the premature N-peptides had run off the gel. The sizes of expressed GLI3^FL^ and GLI3^REP^ in the mutant cell lines (Fig. 2B) were nearly equivalent to (or slightly smaller than) the sizes of WT GLI3^FL^ and GLI3^REP^ proteins. Notably, the unexpected GLI3 protein expression in the mutant cell lines was detected using another anti-GLI3 antibody (Figure S7), thus confirming the reproducibility of GLI3 expression from the out-of-frame mutant alleles.

GLI3^REP^ rather than GLI3^FL^ signals in Fig. 2B provided more conclusive size differences due to the high resolution of the electrophoresis gel. For instance, the size of GLI3^FL^ in 3A11 appeared smaller than in the WT, but other comparisons among GLI^FL^ signals were inconclusive (Fig. 2B). The size of GLI3^REP^ in 3A11 was distinctly the smallest when comparing all of the analyzed samples. Moreover, the GLI3^REP^ signals clearly showed that the exon 2 mutant alleles (2B2 and 2B10) produced larger GLI3 proteins than the exon 3 alleles (3A1, 3A4, 3A8, and 3A11). Illegitimate translation (ITL) may explain the unexpected expression from the out-of-frame indel mutations; thus, we hypothesized that an ITL occurred around the site of the mutations. As shown in Figures S2–S5, several in-frame ATG codons are indeed located in the *Gli3* ORF.

**Figure 3:**
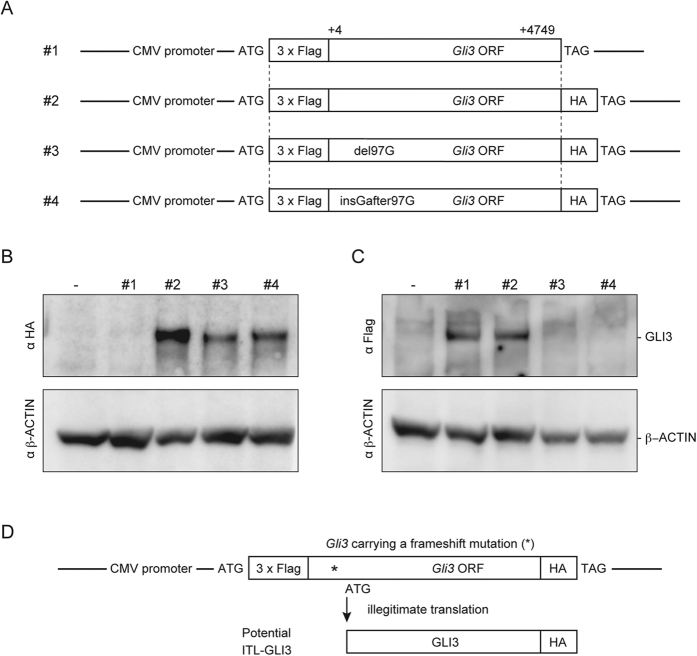
Production of GLI3 protein by ITL *in vitro*. (**A**) Schematic representation of tagged *Gli3* expression vectors. Vector #1 is the single 3xFlag tagged vector. Vectors #2, #3, and #4 are dual-tagged vectors corresponding to the WT, del97G, and insGafter97G, respectively, in Fig. 1B. The *Gli3* ORF contains a fragment from +4 to +4749. Both 3xFlag and HA tags were ligated to the *Gli3* ORF in-frame. (**B** and **C**) Western blotting of transfected cells with anti-HA (**B**), anti-Flag (**C**), and anti-β-ACTIN (**B** and **C**) antibodies. (**D**) Schematic representation of ITL-GLI3 expression from the dual-tagged vectors carrying a frameshift mutation.

To test the above hypothesis, we used four *Gli3* expression vectors with or without tags (Fig. 3A). The 3xFlag and HA tags at the N- and C-termini of GLI3 were used to monitor the initiation and completion of GLI3 in-frame translation, respectively. As shown in Fig. 3B, the GLI3-HA proteins were detected in the transfected NIH3T3 cells with the WT *Gli3* construct (Vector #2) and the two mutant constructs of del97G (Vector #3) and insGafter97G (Vector #4). These findings confirm that the translation reached the end of the original *Gli3* ORF in Vectors #3 and #4, despite the stop codons in exon 2. The 3xFlag-GLI3 was detected in the WT constructs (Vectors #1 and #2), but not in the two mutant constructs (Fig. 3C). As discussed above, the premature N-peptides from Vectors #3 and #4 were not detected in the Western blots. Therefore, the GLI3-HA proteins in the mutant constructs (Lanes #3 and #4 in Fig. 3B) were not translated from the native ATG, but rather from another ATG site. Based on these findings, *Gli3* carrying an out-of-frame indel mutation in either exon 2 or exon 3 should be translated from one of the downstream ATG codons (shown in Figures S2–S5), which leads to the production of an ITL-GLI3 protein (Fig. 3D).

In addition to the observations in NIH3T3 cells, we confirmed the reproducibility of ITL in a different species. The ITL-GLI3 proteins were produced from mutant *Gli3* expression vectors identical to the ones described in Fig. 3A in human HEK293T cells (Figure S8). Thus, ITL is not limited to the mouse NIH3T3 cell line, but rather may universally occur in the presence of a premature termination codon, irrespective of the species.

In order to investigate where the ITL started in Figure S8A by enhancing the resolution of Western analysis to detect slight size differences, we constructed several short dual-tagged expression vectors with the 5′ portion of an 1110-bp fragment from the original ORF (4749 bp), as shown in Figure S9A. We also set several marker expression vectors that initiate the translation from representative ATG codons in the *Gli3* ORF (Figure S9B). As a result, the del97G and insGafter97G vectors clearly expressed ITL peptides with some additional signals. First, the del97G exhibited one unique and two common signals. The unique ITL signal corresponded to marker **b** (Figures S9C and D); thus, the del97G vector initiated ITL from +66. The two additional common signals corresponding to markers **d** and **f** (Figures S9C and D) found in all three of the short expression vectors (Figure S9A) were expressed neither *in vivo* (Fig. 2B) nor in the dual-tagged vector with the full ORF fragment (Figs 3B and S8A). Thus, the del97G mutant allele seemed to initiate ITL from +66 *in vivo*. Likewise, the insGafter97G vector was likely to start ITL from +83 *in vivo* as follows. The insGafter97G vector exhibited two unique signals that corresponded to the marker peptides **a** and **c** (Figures S9C and D) from −11/−5 and +83. Only +83ATG exists *in vivo*, but the −11/−5 ATGs are in a 3xFlag sequence (Figure S9E). The enhanced ITL expression in the short expression vectors (e.g., two common signals and −11/−5 ITL from 3xFlag) is further discussed in Supplementary Discussion 1 and 2. These findings and discussion may suggest that the dual-tagged expression system provides a useful tool to investigate the detailed molecular mechanisms of ITL (Supplementary Discussion 1).

Our findings support the need for the careful design of genome-editing vectors for complete null mutations. For example, the NetStart 1.0 prediction server^14^ can be used to search for potential ITL initiation codons when designing vectors for genome editing. For *Gli3*, in addition to the native initiation codon at +1, there are three in-frame ATG codons at +178, +331, and +352 within 600 bp of the 5′ region (Figures S2–S5). The NetStart 1.0 prediction server predicted that all three of the ATG codons were likely to initiate translation. The common bands corresponding to **d** and **f** found in Figures S9C and D were indeed translated from +178 and +331. Another option to ensure the knockout of the target gene is to eliminate all exons by introducing two DSB/NHEJ sites from the 5′ and 3′ ends to the target coding sequences.

All of the available genome editing technologies, including CRISPR-Cas9, ZFNs^15^,^16^, and TALENs^17^,^18^, introduce DSBs; thus, we strongly recommend surveying any possible ITL initiation site(s) prior to the construction of editing vectors. Notably, we were able to detect endogenous ITL-GLI3 protein using the antibody that was made based on the N-terminal fragment of the protein (Fig. 2A). In this context, the use of an antibody that recognizes the whole (or at least the C-terminus) of the target protein is recommended to examine ITL. Alternatively, *in vitro* assay systems, including the dual-tagged ITL assay system that we developed in this study, may be applicable to pre-examine any unexpected ITL protein expression without preparing any antibodies to the target protein. The dual-tagged ITL assay system instead uses only antibodies against conventional tags.

ITL is neither an exceptional form of gene expression nor an artifact of genome editing. In human diseases, several ITL protein products have been reported. For instance, dominant β-thalassemia was shown to be caused by dominant negative activity of an N-shortened β-globin^7^. In addition, N-shortened RAG1 was reported in Omenn’s syndrome patients with relatively mild immunodeficiency^4^.

ITL has also been reported to regulate the expression of normal genes without mutations. A translation initiation codon is recognized by the ribosomal preinitiation complex through the scanning mechanism from the 5′ end of mRNA^19^. Previous studies showed that almost half of human and mouse transcripts contain uORFs^20^,^21^,^22^. uORFs are small ORFs at the 5′ UTR of mRNA and are thought to regulate the protein expression of the main ORF^9^,^23^. Translation of the main ORF is initiated through various mechanisms^24^,^25^, such as translation reinitiation and leaky scanning. In translation reinitiation, the ribosome that terminates translation of the uORF resumes scanning and reinitiates translation from a downstream ATG. In leaky scanning, the ribosome bypasses the first ATG codon and then initiates translation from a downstream ATG codon. The functional importance of uORFs suggests that translation initiation is an important step in regulation of the expression of many genes. The frameshift mutations established in our study may have generated artificial uORFs (Figures S2–S5). Notably, detailed analysis of the expression studies shown in Figs 2, 3, S8 and S9 may suggest that the molecular mechanism of *Gli3* ITL is leaky scanning (Supplementary Discussion 3). Selection of a translation initiation codon by ribosomes may be more flexible than has been reviewed by others^24^,^25^. Our data instead suggested that the “first-AUG rule” dominated over the strict translation initiation model from “annotated ATG”. Further analysis of the translation of frameshift alleles should make it possible to elucidate the basic mechanisms of ITL.

The data presented here primarily demonstrated the importance of examining any unexpected residual effects of the knockout alleles obtained by genome editing. When utilizing mutant organisms generated with genome editing, this includes not only the off-target mutations but also on-target unexpected gene expression. Furthermore, frameshift mutations induced by genome editing may be useful for translational research on ITL proteins, such as β-globin and RAG1, with respect to molecular diagnoses and/or therapeutic applications.

## Materials and Methods

### CRISPR-Cas9 vector construction

To construct the expression plasmids for sgRNA, target sites against both exon 2 and exon 3 of the mouse *Gli3* gene (Fig. 1B) were selected using the web-based software “CRISPRdirect”^26^. Synthesized double-stranded oligonucleotides were inserted into pSpCas9(BB)-2A-Puro (Addgene plasmid 48139: PX459), in accordance with the protocol published by Ran *et al*.^11^. The following oligonucleotides were used: exon 2, 5′-caccgagatgtcagcgagaaggccg-3′ and 5′-aaaccggccttctcgctgacatctc-3′; exon 3, 5′-caccgctctcatcactagacgtcga-3′ and 5′-aaactcgacgtctagtgatgagagc-3′.

### Tagged vector construction

The previously reported 3xFlag-*Gli3* expression vector^27^ (Vector #1 in Fig. 3A) was used to generate the remaining three dual-tagged vectors shown in Fig. 3A. To construct Vector #2, HA tag was inserted at the 3′ end of the *Gli3* ORF in-frame to Vector #1 by PCR. The following oligonucleotides were used: 5′-aacaggcgtcaggcaatgcc-3′ and 5′-cggaattcctattaagcgtaatctggaacatcgtatgggtaggcctgcataactgcaaggaac-3′. The one-base-pair deletion in the del97G allele and the one-base-pair insertion in the insGafter97G allele (Fig. 1B) were introduced to Vector #2 using the QuickChangeII Site-Directed Mutagenesis Kit (Agilent Technologies) to yield Vector #3 and Vector #4, respectively. The following oligonucleotides were used: del97G, 5′-gatgtcagcgagaagccgtggcctctagta-3′ and 5′-tactagaggccacggcttctcgctgacatc-3′; insGafter97G, 5′-gtcagcgagaagggccgtggcctctag-3′ and 5′-ctagaggccacggcccttctcgctgac-3′. All of the constructs were verified by direct sequencing.

### Cloning of the mutant cell lines using the CRISPR-Cas9 system

NIH3T3 (RCB2767) cells were obtained from the RIKEN BioResource Center, which authenticated the quality of the cell line. NIH3T3 cells were grown in DMEM supplemented with 10% fetal bovine serum, penicillin, and streptomycin under standard conditions. To obtain knockout cell lines carrying mutations in either exon 2 or exon 3, the expression plasmids for sgRNA against exon 2 or exon 3 were independently introduced into NIH3T3 cells. Cells were transfected with Lipofectamine 3000 reagent (Thermo Fisher Scientific Inc.), in accordance with the manufacturer’s instructions. Following puromycin selection, cells were clonally expanded in accordance with the protocol published by Ran *et al*.^11^ with minor modifications. Briefly, 24 hours after transfection, cells were changed to a medium containing 3 μg/μl puromycin and cultured for an additional 48 hours to eliminate non-transfected cells. Puromycin-resistant cells were then trypsinized and cultured in 96-well plates by serial dilutions in order to isolate single cell clones in culture medium without puromycin. One week after plating in 96-well plates, every well in which cells appeared to proliferate from a single cell were identified. We ultimately established eleven independent genome-edited cell lines (Fig. 1B). The cloned cell lines were expanded and subjected to DNA extraction and lysate preparation.

### DNA extraction and mutation analysis

DNA was extracted with the DNeasy Blood & Tissue Kit (Qiagen), in accordance with the manufacturer’s instructions. One of two primer pairs was used to amplify and sequence DNA fragments surrounding the target region of either exon 2 (5′-tttgatggcactgtggtgtt-3′ and 5′-aatttggggtgggagaaatc-3′) or exon 3 (5′-tccacatgatctgagggtga-3′ and 5′-aacacagtcccacggtaagg-3′). On-target small indel mutations in 2B4, 2C6, and 3A4 were detected as homozygous (Fig. 1B); however, these three mutant clones may carry one large deletion allele that included at least one of the primer regions for PCR amplification. We could not determine the mutant alleles from the remaining eight established clones by direct sequencing. These eight heterozygous clones carried two different sequences, which made the sequencing chromatograms overlap. Thus, the PCR products from the eight clones were inserted into a pGEMTeasy vector (Promega), and colony sequencing was performed to determine each allele independently. The inserted 118-bp fragment in 3A8 was identical to a repetitive sequence in pericentromeric regions of the mouse (Figure S5B).

### Western blotting

For Western blotting, the cells were lysed as described by Makino *et al*.^27^. The lysates were then subjected to 6% SDS-PAGE, and immunoblotting was conducted with the SNAP i.d. 2.0 system (Merck Millipore). The primary antibodies were goat anti-GLI3 (AF3690, R&D Systems), rabbit anti-HA (ab9110, Abcam), mouse anti-DDDDK-tag (M185–3S, MBL), and anti-β-actin pAb-HRP-DirecT (PM053-7, MBL). The secondary antibodies were donkey anti-goat HRP (AP180P, Molecular Probes), goat anti-rabbit HRP (ab97051, Abcam), and goat anti-mouse HRP (ab97023, Abcam). All of the original full-sized gel images are shown in Figure S6. All of the Western blotting analyses were completed in duplicate; however, only one result is shown in the figures. According to the manufacturer, the goat anti-GLI3 antibody was made using the region spanning exon 2 to exon 10 of mouse *Gli3* as the antigen (red bar in Fig. 2A).

## Additional Information

**How to cite this article:** Makino, S. *et al*. Illegitimate translation causes unexpected gene expression from on-target out-of-frame alleles created by CRISPR-Cas9. *Sci. Rep.* **6**, 39608; doi: 10.1038/srep39608 (2016).

**Publisher's note:** Springer Nature remains neutral with regard to jurisdictional claims in published maps and institutional affiliations.

## Supplementary Material

Supplementary Materials
