# Supplementary material for: Illegitimate translation causes unexpected gene expression from on-target out-of-frame alleles created by CRISPR-Cas9
Source: Sci Rep. 2016 Dec 21;6:39608. doi: 10.1038/srep39608 (PMC5175197; doi:10.1038/srep39608)
Supplement: Supplementary Materials [file srep39608-s1.pdf]

Illegitimate translation causes unexpected gene expression from on-target out-of-frame alleles created by CRISPR-Cas9

Shigeru Makino, Ryutaro Fukumura, Yoichi Gondo*

Mutagenesis and Genomics Team, RIKEN BioResource Center, 3-1-1 Koyadai, Tsukuba, Ibaraki, 305-0074, Japan

Telephone: +81-29-836-9232

Fax: +81-29-836-9098

*To whom correspondence should be addressed: gondo@brc.riken.jp

Supplementary Discussion

Supplementary Discussion 1: Enhanced ITL expressions in 3xFlag short *Gli3* vectors

All of the short *Gli3* expression vectors of WT, del97G, and insGafter97G (Figure S9A) expressed two common ITL peptides corresponding to markers **d** and **f** (Figures S9C and D). The two common signals did not appear on marker **a** (Figures S9C and D). The marker **a** expression vector is completely identical to the WT vector, except for the 5′ end of the 3xFlag tag sequence. The additional two common signals are thus likely due to the artificial cis-effect of 3xFlag. Notably, the additional two common bands found in the short *Gli3* expression vectors (Figure S9C and D) were never observed with the same dual-tagged expression vectors carrying the 4749-bp full *Gli3* ORF sequence (Figures S6C and S8C). Thus, part or all of the sequence(s) eliminated from the original full 4749-bp ORF was also necessary to suppress the expression of the common additional ITL peptides. On the other hand, the short *Gli3* expression vector without 3xFlag (the GLI3 size markers in Figure S9B) did not express the additional common ITL peptides. Thus, both 3xFlag and elimination of part (or all) of the 4749-bp ORF are necessary for the additional two common ITL expressions. Thus, the replacement of 3xFlag may also be a possible future improvement to eliminate such additional ITL products.

Conversely, 3xFlag with the 1110-bp ORF may be useful to experimentally study translation initiation in vitro by enhancing the very minor ITL expressions. The 3xFlag likely has an artificial cis-effect on both native and ITL translations. Molecular studies on a series of deleted fragments of 4749-bp ORF with 3xFlag may reveal the precise ITL mechanisms by enhancing the ITL phenomenon in vitro.

Supplementary Discussion 2: ITL initiation from 3xFlag sequence

The largest and strongest unique signal in lane insGafter97G (Figures S9C and D) was similar to or slightly larger than that of marker **a**, although the ATG codon of *Gli3* was eliminated in the insGafter97G vector (Figure S9A). Instead, as shown in Figure S9E, four out-of-frame ATG sequences in 3xFlag became in-frame to the 1110-bp ORF in this vector. Thus, -11ATG or -5ATG in 3xFlag seemed to be the initiation site of the ITL of the strong unique peptide in the insGafter97G vector (see 3xFlag+1 frame in Figure S9E). No ATG codons exist in 3xFlag in WT and +2 frame, except at the 5′ end of the original -66ATG. Thus, the largest ITL peptide in the insGafter97G vector was likely initiated from an ATG codon inside the 3xFlag tag.

If we use some other available tags at the 5′ end of the expression vector, such unexpected enhanced ITL effects of 3xFlag may be eliminated. For instance, myc or Strep-tag II does not have any ATG codons in any frame other than the original 5′ end ATG. Indeed, the WT and del97G vectors in Figure S9 did not initiate any ITL products within the 3xFlag sequence at all, since no in-frame ATG codons in 3xFlag existed for these two vectors.

Supplementary Discussion 3: Leaky scanning as a model for Gli3 ITL

In the high-resolution size analysis in Figure S9, we found that the unique signal in the del97G lane, which corresponded to marker **b**, is seemingly expressed from +66ATG (Figure S2). On the other hand, two unique ITL signals were identified in the insGafter97G lane: one was a fainter band corresponding to the +83ATG product (Figure S3) and the other was a larger and stronger signal similar to marker **a** (Figures S9C and D). Thus, all of the three unique ITL bands found in del97G and insGafter97G vectors (Figure S9A) were initiated upstream of the newly created stop codons (Figures S2, S3, and S9E). Only leaky scanning can initiate ITL before the appearance of the stop codon, but the translation reinitiation should occur after the stop codon. In conclusion,

the ITL peptides detected in vivo (lane 2B2 in Figure 2B) are seemingly expressed by leaky scanning.

Supplementary Materials and Methods

Determination of cDNA sequences of the mutant cell line 2B2

The total RNA of the 2B2 cell line was isolated with ISOGEN (Nippon Gene). The isolated RNA was reverse-transcribed with SuperScript III reverse transcriptase (Invitrogen) and oligo$(dT)_{20}$ primers, in accordance with the manufacturer's protocol. The following primers in exon 1 and exon 5 of mouse *Gli3* were used for RT-PCR: 5′-CAGGTCTGTGGATTTGGGAC-3′ (exon1-F) and 5′-GATCCTAATGAAGGGCAAGTC-3′ (exon5-R). For direct sequencing, PCR products were sequenced with either the exon1-F or the exon5-R primer (Figure S1A) using an ABI 3130 Genetic Analyzer (Life Technologies). For colony PCR, the same PCR products were cloned into the pGEM-T Easy vector (Promega), which was then used to transform competent DH5α *E. coli* cells. Next, each cloned cDNA was amplified by the T7 and SP6 primers and sequenced by the exon1-F and exon5-R primers. DNA sequences of a total of 19 colony PCR products were determined (Figure S1B).

Supplementary Figure Legends

Figure S1.

Gli3 cDNA sequences near the mutation sites in biallelic out-of-frame mutant cell line 2B2. (A) We observed no detectable signals of the WT allele in the chromatogram from the direct sequencing of PCR products. (B) In addition, we found no colonies carrying the WT allele in our colony PCR analysis (described in the Supplementary Materials and Methods).

Figure S2.

Putative translation termination and start sites in the del97G allele. The partial nucleotide sequence of mouse *Gli3* ORF (NM008130) from the start codon (+1) to +300 is shown as WT GLI3. The putative premature N-peptide and ITL-GLI3 are shown as del97G-N and del97G-C, respectively. Additional residue shifts caused by the one-base-pair deletion of 97G are shown in blue. Possible ATG sequences acting as an ITL initiation codon for ITL-GLI3 are shown in red. The target region against exon2 is underlined, and the deleted nucleotide of 97G is boxed in red.

Figure S3.

Putative translation termination and start sites in the insGafter97G allele. The partial nucleotide sequence of mouse *Gli3* ORF (NM008130) from the start codon (+1) to +300 is shown as WT GLI3. The putative premature N-peptide and ITL-GLI3 are shown as insGafter97G-N and insGafter97G-C, respectively. Additional residue shifts caused by the one-base-pair insertion of G after 97G are shown in blue. Possible ATG sequences acting as an ITL initiation codon of ITL-GLI3 are shown in red. The target region against exon2 is underlined, and the inserted nucleotide of G is boxed in red.

Figure S4.

Putative translation termination and start sites in the del229A allele. Partial nucleotide sequence of mouse *Gli3* ORF (NM008130) from the start codon (+1) to +420 is shown as WT GLI3. The putative premature N-peptide and ITL-GLI3 are shown as del229A-N and del229A-C, respectively. Additional residue shifts caused by the one-base-pair deletion of 229A are shown in blue. Possible ATG sequences acting as an ITL initiation codon of ITL-GLI3 are shown in red. The target region against exon3 is underlined, and the deleted nucleotide of 229A is boxed in red.

Figure S5.

(A) Putative translation termination and start sites in the insAafter228G allele. The partial nucleotide sequence of mouse *Gli3* ORF (NM008130) from the start codon (+1) to +420 is shown as WT GLI3. The putative premature N-peptide and ITL-GLI3 are shown as insAafter228G-N and insAafter228G-C, respectively. Additional residue shifts caused by the one-base-pair insertion of A after 228G are shown in blue. Possible ATG sequences acting as an ITL initiation codon of ITL-GLI3 are shown in red. The target region against exon3 is underlined, and the inserted nucleotide of A is boxed in red. (B) The inserted sequence of 118 bp in the ins118bpafter228G allele of the 3A8 cell line. The sequences upstream and downstream of the 118-bp insertion are the same as those in the 1-bp insertion of the insAafter228G allele. Putative additional residues (blue color) of the N-peptide and ITL-GLI3 are shown as ins118bpafter228G-N and ins118bpafter228G-C, respectively. A possible ATG sequence acting as an ITL initiation codon of ITL-GLI3 is shown in red.

Figure S6.

Full gel images of Western blots and prestained size marker standards. (A) The full-size images corresponding to those shown in Figure 2B. Chemiluminescence images stained with the indicated antibodies were sequentially captured with a LAS 3000 imaging system. (B) Higher-contrast image of the Western blot shown in Figure S6A. Prestained protein size markers in the transferred membrane are shown on the left to indicate the approximate signal sizes. (C and D) The full-size images of C and D correspond to those shown in Figures 3B and 3C, respectively.

Figure S7.

Confirmation of GLI3 expression in the out-of-frame mutant cell lines with an antibody that recognizes the C-terminal region of GLI3. (A) Schematic representation of the GLI3 protein. The rabbit monoclonal anti-GLI3 antibody (ab181139, Abcam), which was raised against a recombinant fragment of human GLI3 aa 1300–1500 (red bar), was used for Western blotting. Thus, this antibody detects $GLI3^{FL}$ but not $GLI3^{REP}$. (B) Western blotting of lysates from the WT (NIH3T3) and six mutant cell lines carrying biallelic frameshift mutations using the anti-GLI3 antibody. To confirm the specificity of this antibody, lysates of $Gli3^{Xt/Xt}$ embryo brains were used as a control. $Gli3^{XT}$ is a known null allele of *Gli3*[1]. WT and $Gli3^{Xt/Xt}$ embryos were generous gifts from Dr. Gen Yamada at Wakayama Medical University, Japan.

Figure S8.

Confirmation of ITL in the human HEK293T cell line. (A and B) 293T cells were grown in DMEM supplemented with 10% fetal bovine serum, penicillin, and streptomycin under standard conditions. Cells were transfected with the same vectors used in Figure 3 using the

Lipofectamine 3000 reagent, in accordance with the manufacturer's instructions. Western blotting of transfected cell lysates with anti-HA (A), anti-Flag (B), and anti-β-ACTIN (A and B) antibodies. The expression patterns of tagged proteins were almost identical to those observed in mouse NIH3T3 cells (Figures 3B and C). The GLI3-HA proteins were expressed from the WT *Gli3* construct as well as the two mutant constructs. (C and D) The full-size images of C and D correspond to those shown in A and B, respectively. ECL DualVue Western Blotting Markers (GE Healthcare, RPN810) were used to estimate protein sizes (left).

Figure S9.

Analysis of translation start sites of *Gli3* expression vectors carrying del97G and insGafter97G mutations. In order to enhance the size differences, 10% PAGE gels were used with approximately one-quarter (1110 bp) of the full ORF (4749 bp). (A) Schematic representation of dual-tagged expression vectors of the N-terminal fragment of *Gli3* from +4 to +1110, and (B) various "size marker" GLI3 expression vectors. (C, D) Western blotting of transfected HEK293T cells with an anti-HA antibody. The experiment was conducted as shown in Figure S8 with the newly constructed vectors and a high-concentration gel (12% SDS-PAGE). Identical samples were loaded in D and E in a different order to compare the precise band positions between markers and ITL-GLI3 proteins. (E) Positions of ATG codons in the 3xFlag sequence for the interpretation of the result. Only one authentic ATG codon exists at the 5′ end of the 3xFlag (±0 frame). There are four (red color) and no ATG codons in the +1 and +2 frames, respectively. The expected peptide sequences in frames 0, +1, and +2 are shown in black, blue, and gray residues, respectively. An arrow shows the first 6-nucleotide sequence of the *Gli3* ORF from +4 by eliminating the *Gli3* ATG codon.

Reference

1 Maynard, T. M., Jain, M. D., Balmer, C. W. & LaMantia, A. S. High-resolution mapping of the Gli3 mutation extra-toes reveals a 51.5-kb deletion. *Mammalian Genome* **13**, 58-61 (2002).

Figure S1

A

2B2

direct sequencing

+94

WT allele	A A G | G C C G T G G
del97G	A A G | C C G T G G C
insGafter97G	A A G | G G C C G T G

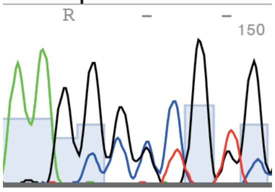

B

Colony PCR

Allele	No. of colonies
WT	0
del97G	8
insGafter97G	11

Figure S2

```
                   10        20        30        40        50        60
          atggaggcccaggcccacagctctacggcgactgagaggaagaaagctgaaaattccatt
WT GLI3   M  E  A  Q  A  H  S  S  T  A  T  E  R  K  K  A  E  N  S  I
del97G-N  M  E  A  Q  A  H  S  S  T  A  T  E  R  K  K  A  E  N  S  I
del97G-C   -  -  -  -  -  -  -  -  -  -  -  -  -  -  -  -  -  -  -  -
                   70        80        90       100       110       120
          gggaaatgtcccacgagaacagatgtcagcgagaaggccgtggcctctagtaccacttcc
WT GLI3   G  K  C  P  T  R  T  D  V  S  E  K  A  V  A  S  S  T  T  S
del97G-N  G  K  C  P  T  R  T  D  V  S  E  K   P  W  P  L  V  P  L  P
del97G-C   -  M  S  H  E  N  R  C  Q  R  E   A  V  A  S  S  T  T  S

                  130       140       150       160       170       180
          aatgaggatgaaagtcctggacagatctatcaccgagagagaagaaacgcaatcactatg
WT GLI3   N  E  D  E  S  P  G  Q  I  Y  H  R  E  R  R  N  A  I  T  M
del97G-N   M  R  M  K  V  L  D  R  S  I  T  E  R  E  E  T  Q  S  L  C
del97G-C  N  E  D  E  S  P  G  Q  I  Y  H  R  E  R  R  N  A  I  T  M

                  190       200       210       220       230       240
          cagcctcagagtgtgcagggtctcaacaaaatcagtgaggagccctcgacgtctagtgat
WT GLI3   Q  P  Q  S  V  Q  G  L  N  K  I  S  E  E  P  S  T  S  S  D
del97G-N   S  L  R  V  C  R  V  S  T  K  S  V  R  S  P  R  R  L  V  M
del97G-C  Q  P  Q  S  V  Q  G  L  N  K  I  S  E  E  P  S  T  S  S  D

                  250       260       270       280       290       300
          gagagggcctcgctgatcaagaaagagatccatggctctctaccacatctggcggagccc
WT GLI3   E  R  A  S  L  I  K  K  E  I  H  G  S  L  P  H  L  A  E  P
del97G-N   R  G  P  R  *  -  -  -  -  -  -  -  -  -  -  -  -  -  -  -
del97G-C  E  R  A  S  L  I  K  K  E  I  H  G  S  L  P  H  L  A  E  P
```

Figure S3

```
                           10        20        30        40        50        60
                   atggaggcccaggcccacagctctacggcgactgagaggaagaaagctgaaaattccatt
WT GLI3            M  E  A  Q  A  H  S  S  T  A  T  E  R  K  K  A  E  N  S  I
insGafter97G-N     M  E  A  Q  A  H  S  S  T  A  T  E  R  K  K  A  E  N  S  I
insGafter97G-C     -  -  -  -  -  -  -  -  -  -  -  -  -  -  -  -  -  -  -  -

                                                    g
                           70        80        90            110       120
                   gggaaatgtcccacgagaacagatgtcagcgagaaggccgtggcctctagtaccacttcc
WT GLI3            G  K  C  P  T  R  T  D  V  S  E  K  A  V  A  S  S  T  T  S
insGafter97G-N     G  K  C  P  T  R  T  D  V  S  E  K  G R  G  L  *  -  -  -
insGafter97G-C     -  -  -  -  -  -  -  M  S  A  R  R  A V  A  S  S  T  T  S

                          130       140       150       160       170       180
                   aatgaggatgaaagtcctggacagatctatcaccgagagagaagaaacgcaatcactatg
WT GLI3            N  E  D  E  S  P  G  Q  I  Y  H  R  E  R  R  N  A  I  T  M
insGafter97G-N     -  -  -  -  -  -  -  -  -  -  -  -  -  -  -  -  -  -  -  -
insGafter97G-C     N  E  D  E  S  P  G  Q  I  Y  H  R  E  R  R  N  A  I  T  M

                          190       200       210       220       230       240
                   cagcctcagagtgtgcagggtctcaacaaaatcagtgaggagccctcgacgtctagtgat
WT GLI3            Q  P  Q  S  V  Q  G  L  N  K  I  S  E  E  P  S  T  S  S  D
insGafter97G-N     -  -  -  -  -  -  -  -  -  -  -  -  -  -  -  -  -  -  -  -
insGafter97G-C     Q  P  Q  S  V  Q  G  L  N  K  I  S  E  E  P  S  T  S  S  D

                          250       260       270       280       290       300
                   gagagggcctcgctgatcaagaaagagatccatggctctctaccacatctggcggagccc
WT GLI3            E  R  A  S  L  I  K  K  E  I  H  G  S  L  P  H  L  A  E  P
insGafter97G-N     -  -  -  -  -  -  -  -  -  -  -  -  -  -  -  -  -  -  -  -
insGafter97G-C     E  R  A  S  L  I  K  K  E  I  H  G  S  L  P  H  L  A  E  P
```

Figure S4

```
                      10        20        30        40        50        60
               atggaggcccaggcccacagctctacggcgactgagaggaagaaagctgaaaattccatt
WT GLI3        M  E  A  Q  A  H  S  S  T  A  T  E  R  K  K  A  E  N  S  I
del229A-N      M  E  A  Q  A  H  S  S  T  A  T  E  R  K  K  A  E  N  S  I
del229A-C       -  -  -  -  -  -  -  -  -  -  -  -  -  -  -  -  -  -  -  -

                      70        80        90       100       110       120
               gggaaatgtcccacgagaacagatgtcagcgagaaggccgtggcctctagtaccacttcc
WT GLI3        G  K  C  P  T  R  T  D  V  S  E  K  A  V  A  S  S  T  T  S
del229A-N      G  K  C  P  T  R  T  D  V  S  E  K  A  V  A  S  S  T  T  S
del229A-C       -  -  -  -  -  -  -  -  -  -  -  -  -  -  -  -  -  -  -  -

                     130       140       150       160       170       180
               aatgaggatgaaagtcctggacagatctatcaccgagagagaagaaacgcaatcactatg
WT GLI3        N  E  D  E  S  P  G  Q  I  Y  H  R  E  R  R  N  A  I  T  M
del229A-N      N  E  D  E  S  P  G  Q  I  Y  H  R  E  R  R  N  A  I  T  M
del229A-C       -  -  -  -  -  -  -  -  -  -  -  -  -  -  -  -  -  -  -  -

                     190       200       210       220       230       240
               cagcctcagagtgtgcagggtctcaacaaaatcagtgaggagccctcgacgtctagtgat
WT GLI3        Q  P  Q  S  V  Q  G  L  N  K  I  S  E  E  P  S  T  S  S  D
del229A-N      Q  P  Q  S  V  Q  G  L  N  K  I  S  E  E  P  S   R  L  V  M
del229A-C       -  -  -  -  -  -  -  -  -  -  -  -  *  G  A  L  A   S  S  D

                     250       260       270       280       290       300
               gagagggcctcgctgatcaagaaagagatccatggctctctaccacatctggcggagccc
WT GLI3        E  R  A  S  L  I  K  K  E  I  H  G  S  L  P  H  L  A  E  P
del229A-N       R  G  P  R  *  -  -  -  -  -  -  -  -  -  -  -  -  -  -  -
del229A-C      E  R  A  S  L  I  K  K  E  I  H  G  S  L  P  H  L  A  E  P

                     310       320       330       340       350       360
               tctctcccttaccgtgggactgtgtttgccatggatccccggaatggctacatggagcct
WT GLI3        S  L  P  Y  R  G  T  V  F  A  M  D  P  R  N  G  Y  M  E  P
del229A-N       -  -  -  -  -  -  -  -  -  -  -  -  -  -  -  -  -  -  -  -
del229A-C      S  L  P  Y  R  G  T  V  F  A  M  D  P  R  N  G  Y  M  E  P

                     370       380       390       400       410       420
               cactaccaccctcctcatcttttccctgccttccatcctcctgtaccaattgatgccaga
WT GLI3        H  Y  H  P  P  H  L  F  P  A  F  H  P  P  V  P  I  D  A  R
del229A-N       -  -  -  -  -  -  -  -  -  -  -  -  -  -  -  -  -  -  -  -
del229A-C      H  Y  H  P  P  H  L  F  P  A  F  H  P  P  V  P  I  D  A  R
```

Figure S5

A

```
                         10        20        30        40        50        60
                 atggaggcccaggcccacagctctacggcgactgagaggaagaaagctgaaaattccatt
WT GLI3          M  E  A  Q  A  H  S  S  T  A  T  E  R  K  K  A  E  N  S  I
insAafter228G-N  M  E  A  Q  A  H  S  S  T  A  T  E  R  K  K  A  E  N  S  I
insAafter228G-C   -  -  -  -  -  -  -  -  -  -  -  -  -  -  -  -  -  -  -  -

                         70        80        90       100       110       120
                 gggaaatgtcccacgagaacagatgtcagcgagaaggccgtggcctctagtaccacttcc
WT GLI3          G  K  C  P  T  R  T  D  V  S  E  K  A  V  A  S  S  T  T  S
insAafter228G-N  G  K  C  P  T  R  T  D  V  S  E  K  A  V  A  S  S  T  T  S
insAafter228G-C   -  -  -  -  -  -  -  M  S  A  R  R  P  W  P  L  V  P  L  P

                        130       140       150       160       170       180
                 aatgaggatgaaagtcctggacagatctatcaccgagagagaagaaacgcaatcactatg
WT GLI3          N  E  D  E  S  P  G  Q  I  Y  H  R  E  R  R  N  A  I  T  M
insAafter228G-N  N  E  D  E  S  P  G  Q  I  Y  H  R  E  R  R  N  A  I  T  M
insAafter228G-C   M  R  M  K  V  L  D  R  S  I  T  E  R  E  E  T  Q  S  L  C

                                                                  a
                        190       200       210       220         v       240
                 cagcctcagagtgtgcagggtctcaacaaaatcagtgaggagccctcgacgtctagtgat
WT GLI3          Q  P  Q  S  V  Q  G  L  N  K  I  S  E  E  P  S  T  S  S  D
insAafter228G-N  Q  P  Q  S  V  Q  G  L  N  K  I  S  E  E  P  S  N V  *  -  -
insAafter228G-C   S  L  R  V  C  R  V  S  T  K  S  V  R  S  P  RT  S  S  D

                        250       260       270       280       290       300
                 gagagggcctcgctgatcaagaaagagatccatggctctctaccacatctggcggagccc
WT GLI3          E  R  A  S  L  I  K  K  E  I  H  G  S  L  P  H  L  A  E  P
insAafter228G-N   -  -  -  -  -  -  -  -  -  -  -  -  -  -  -  -  -  -  -  -
insAafter228G-C  E  R  A  S  L  I  K  K  E  I  H  G  S  L  P  H  L  A  E  P

                        310       320       330       340       350       360
                 tctctcccttaccgtgggactgtgtttgccatggatccccggaatggctacatggagcct
WT GLI3          S  L  P  Y  R  G  T  V  F  A  M  D  P  R  N  G  Y  M  E  P
insAafter228G-N   -  -  -  -  -  -  -  -  -  -  -  -  -  -  -  -  -  -  -  -
insAafter228G-C  S  L  P  Y  R  G  T  V  F  A  M  D  P  R  N  G  Y  M  E  P

                        370       380       390       400       410       420
                 cactaccaccctcctcatcttttccctgccttccatcctcctgtaccaattgatgccaga
WT GLI3          H  Y  H  P  P  H  L  F  P  A  F  H  P  P  V  P  I  D  A  R
insAafter228G-N   -  -  -  -  -  -  -  -  -  -  -  -  -  -  -  -  -  -  -  -
insAafter228G-C  H  Y  H  P  P  H  L  F  P  A  F  H  P  P  V  P  I  D  A  R
```

B

```
118bp insertion        catttctcattttttcacgttttttagtgatttcatcatttttcaagtcgtcaagtggatg
ins118bpafter228G-N    H  F  S  F  F  T  F  F  S  D  F  I  I  F  Q  V  V  K  W  M
ins118bpafter228G-C  R  I  S  H  F  S  R  F  L  V  I  S  S  F  F  K  S  S  S  G  C

ins118bpafter228G      tttctcattttccatgattttcactttttcttgccatattccacgtcctacagtggaca
ins118bpafter228G-N    F  L  I  F  H  D  F  H  F  S  C  H  I  P  R  P  T  V  D
ins118bpafter228G-C     F  S  F  S  M  I  F  T  F  L  A  I  F  H  V  L  Q  W  T
```

Figure S6

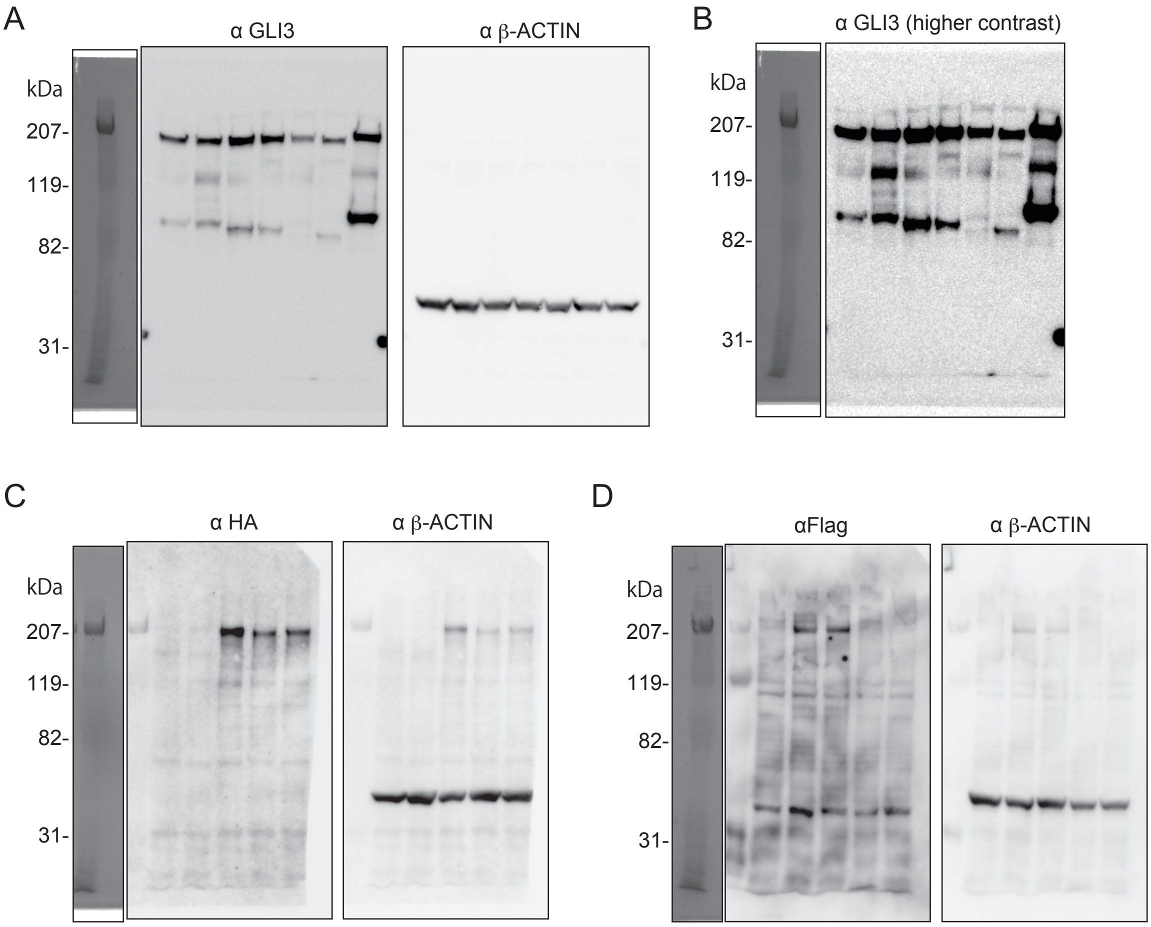
A
α GLI3
α β-ACTIN
kDa
207-
119-
82-
31-
B
α GLI3 (higher contrast)
kDa
207-
119-
82-
31-
C
α HA
α β-ACTIN
kDa
207-
119-
82-
31-
D
αFlag
α β-ACTIN
kDa
207-
119-
82-
31-

Figure S7

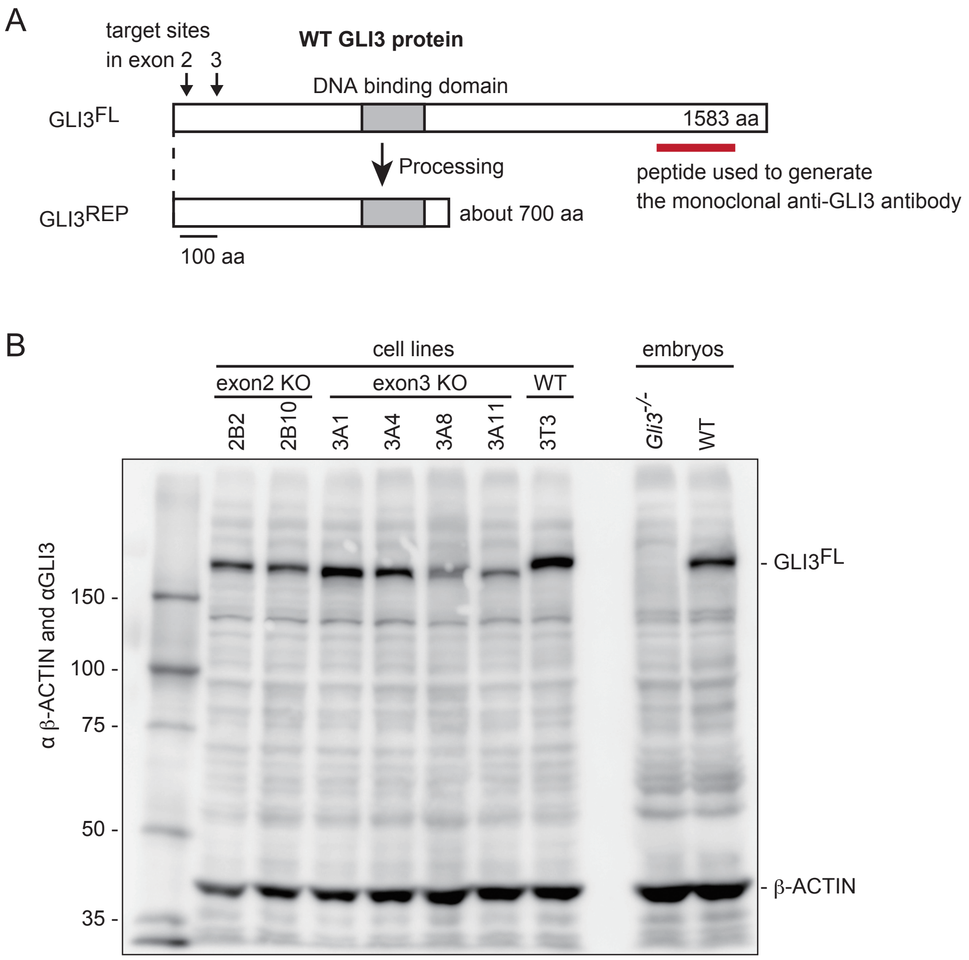
A
target sites
in exon 2 3
WT GLI3 protein
DNA binding domain
GLI3FL
1583 aa
Processing
peptide used to generate
the monoclonal anti-GLI3 antibody
GLI3REP
about 700 aa
100 aa
B
cell lines
embryos
exon2 KO
exon3 KO
WT
2B2
2B10
3A1
3A4
3A8
3A11
3T3
Gli3-/-
WT
α β-ACTIN and αGLI3
150 -
100 -
75 -
50 -
35 -
- GLI3FL
- β-ACTIN

Figure S8

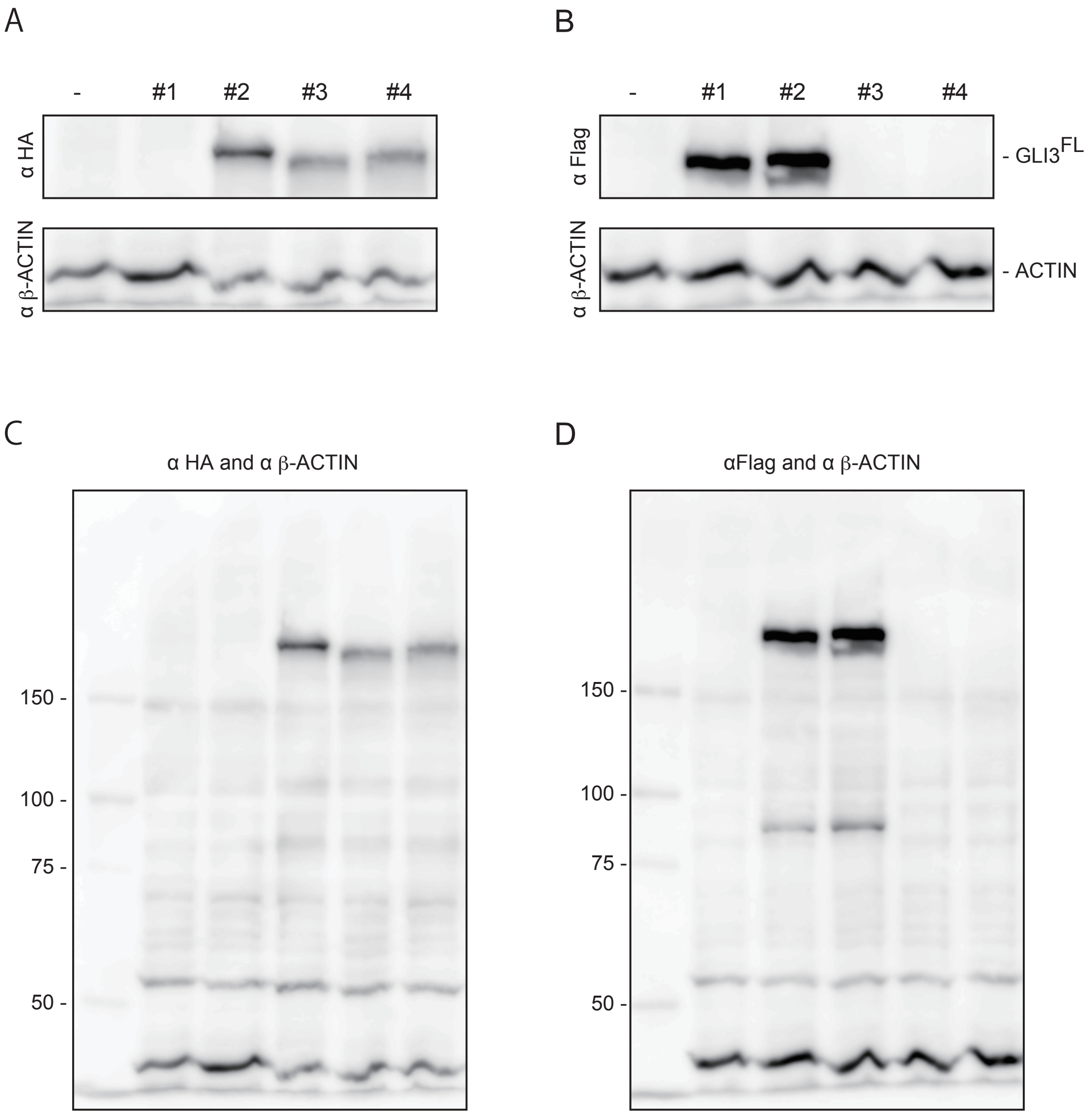
A
-
#1
#2
#3
#4
α HA
α β-ACTIN
B
-
#1
#2
#3
#4
α Flag
α β-ACTIN
- GLI3FL
- ACTIN
C
α HA and α β-ACTIN
150 -
100 -
75 -
50 -
D
αFlag and α β-ACTIN
150 -
100 -
75 -
50 -

Figure S9

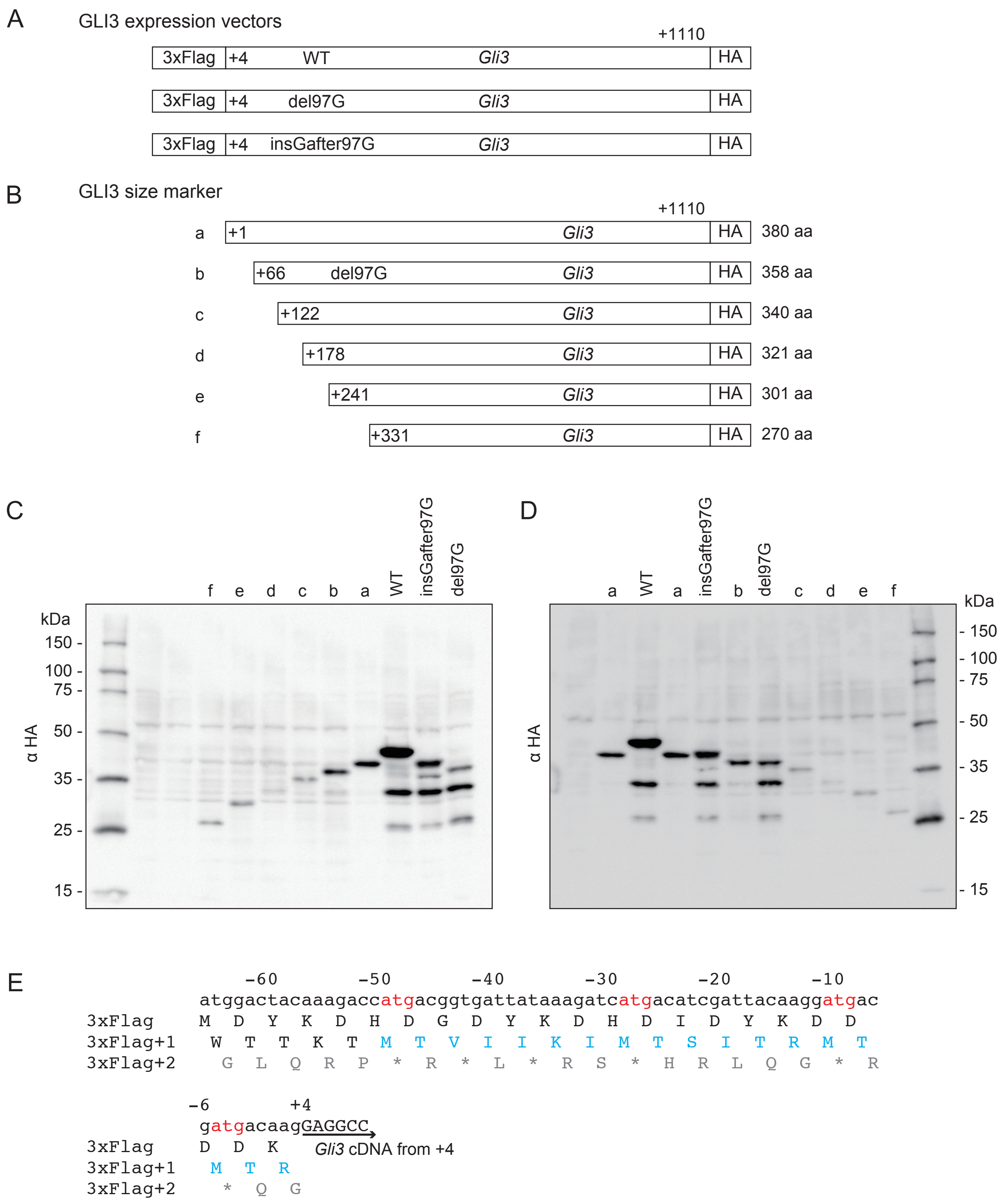
A
GLI3 expression vectors
+1110
3xFlag +4 WT Gli3 HA
3xFlag +4 del97G Gli3 HA
3xFlag +4 insGafter97G Gli3 HA
B
GLI3 size marker
+1110
a +1 Gli3 HA 380 aa
b +66 del97G Gli3 HA 358 aa
c +122 Gli3 HA 340 aa
d +178 Gli3 HA 321 aa
e +241 Gli3 HA 301 aa
f +331 Gli3 HA 270 aa
C
f e d c b a WT insGafter97G del97G
kDa
150 -
100 -
75 -
50 -
35 -
25 -
15 -
α HA
D
a WT a insGafter97G b del97G c d e f
kDa
- 150
- 100
- 75
- 50
- 35
- 25
- 15
α HA
E
-60 -50 -40 -30 -20 -10
atggactacaaagaccatgacggtgattataaagatcatgacatcgattacaaggatgac
3xFlag M D Y K D H D G D Y K D H D I D Y K D D
3xFlag+1 W T T K T M T V I I K I M T S I T R M T
3xFlag+2 G L Q R P * R * L * R S * H R L Q G * R
-6 +4
gatgacaagGAGGCC
3xFlag D D K
Gli3 cDNA from +4
3xFlag+1 M T R
3xFlag+2 * Q G
